# Editorial: Hematophagous arthropod saliva: a multifunctional tool

**DOI:** 10.3389/fcimb.2022.977511

**Published:** 2022-07-13

**Authors:** Regis Gomes, Iva Kolářová, Anderson Sá-Nunes, Matheus Carneiro

**Affiliations:** ^1^ Biotechnology, Oswaldo Cruz Foundation, Eusébio, Brazil; ^2^ Laboratory for Biology of Insect Vectors, Department of Parasitology, Faculty of Science, Charles University, Prague, Czechia; ^3^ Laboratory of Experimental Immunology, Department of Immunology, Institute of Biomedical Sciences, University of São Paulo, São Paulo, Brazil; ^4^ National Institute of Science and Technology in Molecular Entomology, National Council of Scientific and Technological Development, Rio de Janeiro, Brazil; ^5^ Snyder Institute for Chronic Diseases, University of Calgary, Calgary, Canada

**Keywords:** saliva, hematophagous vectors, bioactive molecules, immunomodulation, immunotherapy

## Introduction

In recent years, an increasing body of evidence shed light on the importance of salivary proteins from hematophagous arthropods. Although the first studies on the cutaneous reactions to mosquito bites in humans date back about a century (Gordon, 1922; Boycott, 1928), it took many decades for hematophagous arthropod saliva to be recognized as a source of bioactive molecules on host hemostasis and immunity (Ribeiro, 1995). Several groups demonstrated that the salivary secretion of blood feeding vectors could enhance pathogen transmission and/or establishment. This phenomenon, named saliva-assisted transmission (Nuttall, 2018), was initially proposed for ticks and later expanded to other hematophagous arthropods. These findings created several lines of investigation now involving the hematophagous vector-pathogen-vertebrate host triangle (Gomes and Oliveira, 2012). In addition, several groups have been also working with the concept of saliva-based vaccines, showing promising results in different models of vector-borne diseases (Manning et al., 2018; Sá-Nunes & Oliveira, 2021).

Due to the limitation of dissecting the whole salivary glands or direct collecting of pure saliva, the “omics” approaches represented a revolution to the research field on saliva from blood-feeding arthropods. Thus, proteomes, transcriptomes and genomes of hematophagous arthropods provided a huge catalog of potential new molecules to be explored. This led to the discovery and development of saliva-derived biotherapeutic molecules useful in different clinical settings. The 4 articles published on this especial topic bring some of the new discoveries, putting important pieces of puzzle into the picture helping us to understand the complex interaction between saliva-parasite-host and its further use in human/patients benefit.

In a minireview by Aoki et al. the readers can learn how sand fly saliva can act as multifunctional bioactive molecules. Many studies reveal the potential use of sand fly salivary proteins for the control of Leishmaniasis, either as anti-Leishmania vaccines or as biomarkers of vector exposure (Valenzuela et al., 2001; Gomes et al., 2012; Grespan et al., 2012; Lestinova et al., 2017). Nevertheless, Aoki et al. also stressed out that some sand fly salivary proteins were identified as triggering autoimmunity in humans. The development of anti-sand fly saliva antibodies that cross-react with the ectodomain of human desmoglein 1 (Dsg1), is associated with skin autoimmune diseases, such as Pemphigus. Previous work from this group demonstrated a possible cross-reactivity between IgG4 and IgE antibodies against the sand fly *Lutzomyia longipalpis* LJM17 and LJM11 proteins with human Dsg1. Moreover, some sand fly salivary proteins (e.g., *Phlebotomus papatasi* PpSP32) can induce immunocomplexes with desmogleins, triggering Pemphigus in genetically predisposed individuals. Therefore, we should bear in mind that salivary proteins might elicit autoimmune response and thus every vaccine candidate should be carefully tested for such side effects.

One of such vaccine candidates was introduced in an interesting approach by Lajevardi et al. They showed a multivalent live vaccine against cutaneous leishmaniasis using *Leishmania tarentolae* as a vector co-expressing PpSP15 and PsSP9 salivary proteins plus CpG as an adjuvant. BALB/c mice were immunized and challenged with two different parasites, *Leishmania major* and *Leishmania tropica*, resulting in a protective Th1 immunity. Importantly, this is the first study testing a live vaccine based on the combination of two different salivary proteins that protect against two different species of *Leishmania*, opening a new pathway for development of a safe saliva-based vaccine combining different salivary antigens.

Carvalho-Costa et al. further explore the salivary glands and intestine omics from the triatomine *Rhodnius neglectus*, a potential vector of *Trypanosoma cruzi*, giving an important contribution in this area. The authors analysed the global transcriptional genes of salivary glands and intestines of this triatomine during fasting, in blood fed and in blood fed plus *T. cruzi.* In summary, blood plus parasite inhibited the expression of blood processing genes involved in insect metabolism (e.g., Antigen-5 precursor, Pr13a, and Obp), detoxification (Sult1) in the intestine and acid phosphatases in the saliva. They also demonstrated a decrease of lipocalins and nitrophorins expression in salivary glands and the presence of two new proteins in the intestine, named as pacifastin and diptericin. Importantly, several transcripts of unknown proteins with potentially interesting function were found in the saliva and intestine. The results suggest that the parasite can change the transcriptomic profile, thereby contributing to our understanding of parasite-vector interactions and opening new directions to investigate the network between feeding physiology and post-meal/infection in triatomines.

In an elegant study, Praça et al. used high-throughput transcriptomics and proteomics to report the first sialome study on the synanthropic triatomine *Triatoma sordida*. The authors sequenced 57,645,372 reads that were assembled into 26,670 coding sequences. From these, a total of 16,683 were successfully annotated. Interestingly, sialotranscriptomic profile shows lipocalin as the most abundant protein family within putative secreted transcripts, also demonstrated in the aforementioned study by Carvalho-Costa et al. Trialysins and Kazal-type protease inhibitors were highlighted followed by ubiquitous protein families and several enzyme classes. The proteomics further identified 132 proteins in *T. sordida* salivary gland soluble extract, including lipocalins, Hemiptera-specific families of proteins, CRISP/Antigen-5 and Kazal-type inhibitors. Having the sialome of *T. sordida* in hand, the next step could be to explore the immunopharmacological activities of these molecules.

There is a great interest in the development of new immunomodulators, vaccines, anti-inflammatory, and anti-hemostatic drugs, in addition to markers of exposure for hematophagous vectors based on their salivary proteins. The articles published in this Research Topic highlight several important aspects that can effectively select promising candidates, help understanding vector’s physiology and vector-host interactions, as well as the evolutionary mechanisms leading to the insect’s adaptation to the blood-feeding behavior. The articles clearly show the advantage of the “omics” approach that represent state-of-the-art technique in the research of saliva from blood-feeding arthropods, providing catalogues of potential new molecules to be explored *in silico* and in biological platforms. We hope that more research groups will further invest their effort in this topic, exploring also other hematophagous arthropods trying to understand the natural interaction of vector-host-pathogen in its whole complexity.

## Author contributions

All authors listed have made a substantial, direct, and intellectual contribution to the work and approved it for publication.

## Conflict of interest

The authors declare that the research was conducted in the absence of any commercial or financial relationships that could be construed as a potential conflict of interest.

## Publisher’s note

All claims expressed in this article are solely those of the authors and do not necessarily represent those of their affiliated organizations, or those of the publisher, the editors and the reviewers. Any product that may be evaluated in this article, or claim that may be made by its manufacturer, is not guaranteed or endorsed by the publisher.
